# Circular Approaches in Small-Scale Food Production

**DOI:** 10.1007/s43615-021-00129-7

**Published:** 2021-11-22

**Authors:** Petra Schneider, Vincent Rochell, Kay Plat, Alexander Jaworski

**Affiliations:** 1 Magdeburg-Stendal University of Applied Sciences, Breitscheidstr. 2, D-39114 Magdeburg, Germany; 2 Leipzig University, AG Greenhub, Philipp-Rosenthal-Str. 55 (SIKT), D-04103 Leipzig, Germany

**Keywords:** Traditional food production, Small-scale food production, Circular gardening, Integrated agroecology, Urban farming, Controlled environment agriculture

## Abstract

Globally, food production is one of the main water and energy consumers. Having in view the growing population on global scale, a higher efficiency of food production is needed. Circular approaches offer a large potential to enhance the efficiency of food production and have a long tradition in the food production process of mankind. However, industrial farming has interdicted traditional cycle-closed farming approaches leading to a variety of environmental challenges. The contribution illustrates the basics of traditional gardening and farming approaches and describes how their characteristics are adapted in innovative modern farming systems like aquaponic, permaculture, urban farming, as well as recovered traditional farming systems. The approach to combine traditional farming methods with modern ones will provide multiple benefits in the future to ensure food security. There is to be underlined that such a strategy holds a substantial potential of circular flux management in small scale food production. This potential could be transposed to a larger scale also, particularly in terms of agroforestry and integrated plant and animal husbandry or integrated agriculture and aquaculture. In this way, small-scale food production holds a large potential for the future implementation of the water-energy-food security nexus.

## Introduction

When implementing the global 2030 Agenda for Sustainable Development to achieve the Sustainable Development Goals (SDGs), particularly SDG 2 that calls for an end to hunger by 2030, the questions of how food security can be ensured, sustainability in agriculture plays a central role. The right to food is a human right. All governments have a duty to implement this right, which is anchored in international law. Nevertheless, as per the Food and Agriculture Organization of the United Nations (FAO), a minimum of around 820 million people (11%) worldwide were chronically under-nourished in 2018 [1].

According to the United Nations’ Committee on World Food Security, food security is defined as the means that all people, at all times, have physical, social, and economic access to sufficient, safe, and nutritious food that meets their food preferences and dietary needs for an active and healthy life (https://www.ifpri.org, accessed 22–02-2021). In theory, global food production is already sufficient to feed twelve billion people. According to (FAO), 1,800 kilocalories are the threshold value for a healthy life. If an adult eats less for a longer period of time, this is considered starvation. However, the distribution of the available calories is very different around the world. While a person in Europe has an average of approx 3,400 kcal/d*cap, in Africa it is only approx. 2,600 kcal/d*cap. In several regions and population groups, the supply is far below that (source FAOstats database (food balance sheets, http://www.fao.org/faostat/en/#data/FBS, accessed 22–02-2021). The reasons for this are very diverse, for instance, natural conditions such as climate or the nature of the soil. The unequal distribution of food also has an ecological dimension: Food production needs resources, and they are not available in unlimited quantities. How much and which resources are required differs depending on the type of food and the production conditions.

The basic resources are water and energy. Water resources are very unevenly distributed around the world. At the same time, the water demand for production of different types of local food is extremely different. A third fundamentally important resource is agricultural land or soil. Increasing agricultural production is often accompanied by an increase in the area under cultivation. In many regions of the world, increasing demand for land is leading to the clearing of forests. According to the German Environmental Agency (2015) [2], in total almost ten million hectares of arable land are lost every year. Other causes for this development are overfertilization, intensive agriculture, and the cultivation of monocropping, which always deprive soils of the same nutrients and therefore permanently damage them [3–5]. In addition, the land expansion is limited because feed production already binds a third of the arable land available worldwide [6]. One approach to tackle the many environmental problems is the orientation towards the principles of a more sustainable agriculture that uses soil preserving methods and acts without synthetic chemical agents. On the other hand, weeds are fought mechanically, organic fertilisers are used, and the use of antibiotics is largely dispensed in animal husbandry. In addition, the most closed operational nutrient cycle should be achieved, that means that feed and fertilisers are produced as far as possible on the farms where they are used. A global environmentally sustainable and resource-efficient agriculture requires a shift to fully organic agriculture within a properly designed food system. The conversion to organic alone would not lead to the desired effects for sufficient food production by 2050 [7].

Part of a well-designed food system to tackle food security is the approach of fostering small-scale food production and preservation of traditional systems in practice. Small-scale food production is as old as humanity. In many regions of the world, it still plays a substantial role for food supply. Traditional systems tend to be in harmony with natural closed cycles. Often it is a small garden or a small agricultural area that is used for self-sufficiency. Khalil et al. (2017) [8], on behalf of FAO, considered small-scale food producers as smallholders and concluded that they are taking the bottom 40% of the (i) operated land size, (ii) the tropical livestock units, and (iii) the distribution of revenues. This definition underlines that small-scale food production is particularly relevant in the developing countries of the Global South. Other definitions refer to garden or farm size. As Khalil et al. (2017) [8] stated, based on the research of Eastwood et al. (2009) [9], about 70% of the literature defines smallholders in terms of the physical size of the farm, basically in terms of hectares of operated or owned land by individual farmers and their families. Thapa (2009) [10] concluded that the most common measure is farm size, usually referring to small farms with less than 2 ha of cropland.

The understanding of small-scale food production in the industrialised countries is different and takes into consideration that those countries usually have a highly efficient industrial agriculture and do not depend on small-scale food production. However, industrial agriculture is very often connected with long-lasting environmental impacts like loss of nutrients, which is often mitigated by applying artificial fertilisers as well as large-scale application of pesticides for pest control [11, 12]. Small-scale gardening has a long tradition in industrialised countries, dating back 200 years ago as allotment gardens [13]. In that period, allotment gardens were developed as a plot of communal land for the poor where families could partially cover their food needs [14]. In the last decades, allotments experienced a great renaissance in terms of “sustainable food” or “local food strategies” as part of sustainable urban development and were transformed into the main leisure and recreational facility in the urban realm that serves social interaction [15]. Typical sizes of allotment gardens are in the range of 100 to 1000 m^2^, as illustrated in several sources. For instance, in Germany allotment gardens are limited to 400 m^2^ (regulated by law [16]); in Norway they have a typical size of 150–300 m^2^ [17]; in The Netherlands and the Czech Republic, their size is between 100 and 500 m^2^ [18], moreover, 200 m^2^ in Sambia [19], up to 2.5 ha in South Africa [20], and up to 3 ha on the Indonesian islands [19].

The scope of the study is a review on small-scale food production approaches in developing and developed countries to identify the potential for circular approaches of water, nutrients, and energy. The contribution illustrates the basics of traditional gardening and farming approaches and describes how their characteristics are adapted in innovative modern farming systems. In view of the growing world population, the goal for the future is to achieve more agricultural productivity while avoiding negative effects on the environment.

## Methodology

The methodology adopted in this study is based on following steps:

- Literature research through the Web of Science and other scientific research platforms like Scopus and Google Scholar in terms of traditional and modern small-scale food production systems
- Data collection regarding material flows, particularly for water, nutrients, and energy
- Results of own investigations on innovative small-scale food production approaches from investigations at the institutions of the authors
- Visualisation and illustration of the material flows through the STAN (short for subSTance flow ANalysis) freeware for performing material flow analysis according to the Austrian standard ÖNorm S 2096 [21], current version STAN 2.6 (2017)
- Assessment of the identified small-scale food production approaches through a SWOT analysis, where S stands for strengths, W for weaknesses, O for opportunities, and T for threats [22, 23]
- Drawing of conclusions in terms of circular loop handling based on the foregoing.

The reference to the small-scale food production system size is important for the illustration of the material flows and hence the productivity assessment. We are aware that traditional and modern systems cannot be compared in terms of material flows; however, the overall scope is to illustrate the small-scale food production schemes, to identify the potential for circular economy approaches, and to find out sustainability potential for the future that can be concluded from the experiences of the past.

## Literature Review Results—Overview on Small-Scale Food Production

### A Short Look on History

The review results were structured in two parts: traditional and modern farming approaches. At some point in human history, the development of agriculture and sedentarism seemed inevitable. In at least half a dozen regions on earth (Middle East, Indus Valley, etc.) animals were domesticated and different plants were cultivated. This led to a better food supply. Archaeologists discovered the first evidence of agricultural methods in the area of the “Fertile Crescent”, the region that stretches in a wide arc from the Persian Gulf in the east through southern Turkey to Israel in the west. Eleven thousand years ago, there were ideal conditions for growing grain here. Furthermore, also aquaculture that is used to breed aquatic organisms was developed in China and India empirically in fresh water already in 1500 B.C.E. [24]. One thousand years later, the Etruscans and Romans systematically farmed oysters and fish in lagoons on the Mediterranean [25]. In the Middle Ages, pond breeding made carp a “domestic animal”; there are also isolated mussel breeding methods, the technology of which hardly changed until the twentieth century. The traditional farming fundamentals are still in use nowadays.

### Traditional Small-Scale Farming Systems

#### Traditional Gardening and Farming

Forms of cover crop cultivation, intercropping, crop rotation, and shifting cultivation (also called swidden cultivation) are known on all continents. Shifting cultivation refers to a technique of rotational farming in which land is cleared for cultivation (normally by fire) and then left to regenerate after a few years.

Crop rotation describes the cultivation of crops in a sequence on the same land. It offers a solution to the declining soil quality caused by monocultures and can have positive effects on yields and the use of resources [26].

Intercropping describes the cultivation of two or more plant species in the same field [27]. With optimal implementation, through the right choice of compatible plants and a well thought-out model, cover crop cultivation enables positive effects on agriculture by using natural cooperation between plant species [27–29]. In this way, improved soil quality, resource efficiency, plant productivity, as well as greater resistance of the agro-ecosystem to pests and climate-related crop failures can be achieved. In addition, biodiversity can be increased, and an alternative to monocultures can be created [26, 28]. In Mozambique, intercropping was investigated using local smallholder practices and led to the conclusion that there is the potential to reduce the risk of crop failure and to increase production [30]. Cover crop cultivation refers to crops grown to cover the soil in order to have positive effects on the agricultural system such as less soil erosion and nutrient losses [26]. The term describes plants that are grown for ecological reasons and are not used as crops [31]. In this case, cover crops can provide a living mulch for the crops [26, 31]. In addition to erosion protection and increased nutrient content in the soil, cover crops can control pests and weeds, fix carbon, and reduce runoff or store water [26, 31, 32].

Urban gardening and urban agriculture have existed since the emergence of cities [24, 25]. The phenomenon of urban cultivation can be observed globally as a widespread practice. The advantages include the production of fresh field crops and vegetables, the resulting improved food security, and sustainable livelihoods. Especially in economically uncertain times or phases of undersupply, they traditionally represent a possibility of survival [33]. For example, urban horticulture boomed after both world wars in view of the severe damage and lack of accommodation in the city of Berlin [34]. Even today, great importance can be assigned to urban agriculture in some megacities in industrialised countries. For example, in Shanghai with its 24 million inhabitants, 60% of the vegetables consumed and 90% of the eggs consumed are produced within the city limits [35]. This state of affairs may represent the future of many urban areas, as forecasts indicate that urbanisation will continue to increase rapidly over the next few decades.

#### Agroforestry

**Fig. 1:**
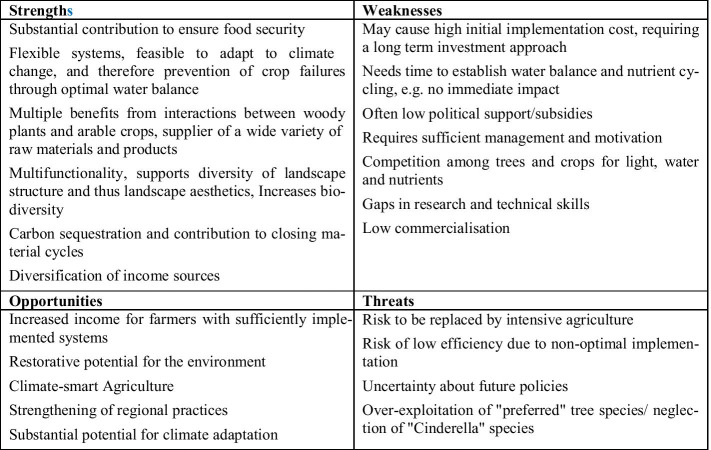
SWOT analysis of agroforestry systems

The term agroforestry includes land use systems in which perennial wood plants are cultivated on the same area together with useful plants and/or livestock [36]. The aim of these systems is to bring about a positive interaction between the individual components, both ecologically and economically [36]. Nowadays, agroforestry systems are most widespread in tropical regions such as South-East Asia, Latin and Central America, and in the areas of sub-Saharan Africa, where they are often practised by smallholders. A variety of traditional practices can be found here [26]. It is estimated that around 10 million km^2^ of land are used for agroforestry nowadays [37]. Lorenz and Lal (2018) [37] grouped these systems into agrosilvicultural (crops and trees), silvopastoral (pasture/animals + trees), and agrosilvopastoral (crops + pasture/animals + trees). In the past few decades, numerous indigenous forms of agro-forestry have been introduced into the field of modern scientific land use scenarios [38]. Traditional land management practices were included into the design of modified systems and technologies [38]. The result are “improved” systems for both tropical and temperate climates [37]. Thus, in the recent past, agroforestry systems in the form of windbreak hedges and alley cropping systems have been used successfully in North America and Central Europe [39–41]. According to Lorenz and Lal (2018) [37], major agroforestry systems in tropical regions comprise alley cropping, home gardens, improved fallows, multipurpose trees, silvopasture, shaded perennial crop systems, shelterbelts/windbreaks (hedges), and taungya (originating in Burma, [42]). The applied systems in temperate regions are alley cropping, forest farming, riparian buffer stripes, silvopasture, and windbreaks [37]. Conventional systems such as alley cropping (integrating fast-growing woody species in grain fields) or home gardens (combining different trees and useful plants and sometimes cattle on farms) are in part successfully tested, investigated, and practised, particularly in the tropics [37]. An overview on the strengths and weaknesses of the system approach is given in Fig. 1.

**Fig. 2:**
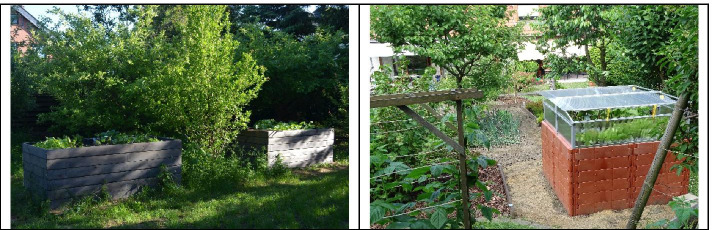
Modern forms of small-scale agroforestry: raised beds in orchards, impressions from Germany (photo credit: Petra Schneider)

Through the process of photosynthesis, trees bind carbon and store excesses as above and underground biomass and as organic carbon in the soil [37]. In addition, wood for energetic use can be obtained from agroforestry and thus form an alternative to fossil fuels [43]. In an example scenario according to Böhm, in which half of the arable land in Germany would be used with agroforestry systems with a tree share of 10%, 10 million tons of CO_2_ eq/a could be bound in wood mass [44]. Moreover, agroforestry may also improve biodiversity by creating structural diversity and creating retreats for animals (Grünewald et al. 2009). However, traditional agroforestry is at risk in many countries because it is being displaced by other farming methods [45]. Integrated animal husbandry in agroforestry does not only supply farmers with milk and meat, but also recycles their feed into fertiliser (Fig. 2). A traditional example from Europe is the Dhesa system from Spain. Here, areas under the forest canopy are cleared through by grazing in order to create arable land [26]. Depending on the system used, the feed intake can largely consist of crop residues from fallow fields and integrated trees. In Botswana, an estimated 25% of feed could come from trees and shrubs [46]. From an economic point of view, the implementation of woody plants can not only prevent harvest collapses through their protective effect, but also achieve greater efficiency in land use.

#### Aquaculture

According to Marshall (2017), aquaculture describes the rearing and harvesting of aquatic organisms such as fish, crustaceans, molluscs, and aquatic plants under controlled conditions. This implies intervening in the rearing process in any form, such as feeding or protection from enemies [25]. A traditional form of aquaculture that has been preserved to this day is the VAC system from Vietnam, in which fish ponds were integrated into a multifunctional system [47]. Traditional fish farming is practised almost exclusively in developing countries. According to Bhujel [48], the largest share is present in Asia.

Aquaculture in ponds is widespread around the world. The intensity with which it is operated varies from extensive low-input systems to optimised systems with improved water exchange, high-quality feed, adapted fish species, and other professional practices. The water requirement plays an important role in pond systems. If there is too much evaporation and seepage, ponds are not practical for fish farming. If these parameters do not pose a risk, ponds can also be used for water retention and irrigation for agriculture [24].

Cage breeding is often carried out on a large scale, for example, as mariculture in the sea, often associated with severe environmental risks [25]. As a small-scale method, operated by fishermen or family-run farms, traditional baskets made of wood and bamboo or modern synthetic materials are sometimes still used [49–51]. Due to their flexibility, easy installation and maintenance, low labour requirements, and simple operation, cage fish farming has become usual among resource-poor smallholders. However, in this system, fish are also more susceptible to diseases caused by crowded fish populations or poor water quality. Since a lot of feed escapes from the cages into the water, the animals have to be fed several times a day. Together with high densities, this can cause a risk of reduced oxygen and increased ammonia concentration in the surrounding water [50, 51].

Recirculating aquaculture systems (RAS) embodies a strategy to intensify fish production while at the same time reducing waste [52]. For this purpose, water used is recycled by cleaning processes removing fish waste and food residues, neutralising ammonia, and enriching oxygen so that the clean water can be reused for breeding [25]. In addition to improved waste management, this also reduces water consumption [53]. Furthermore, recirculating aquacultures offer the possibility of fish farming in otherwise unsuitable environments, such as dry areas or urban areas. High upfront investments and operating costs are offset by advantages such as low space requirements, high stocking densities of fish, and easier treatment of disease outbreaks [25]. Due to high energy, labour, and feed costs, there are difficulties in up-scaling the recirculating aquaculture, which is why most farms operate on a small scale, up to 50 tons per year [52].

For all aquaculture systems, the use of marine fish meal and oil can cause environmental issues. Some species grown in aquaculture need high amounts of protein in their nutrition. Even though fish show an efficient protein conversion, in total a lot of small pelagic fish are captured from the oceans to be converted into fish feed. However, the number of these organisms is limited, and overfishing can have a negative impact on the marine food chain [54].

**Fig. 3:**
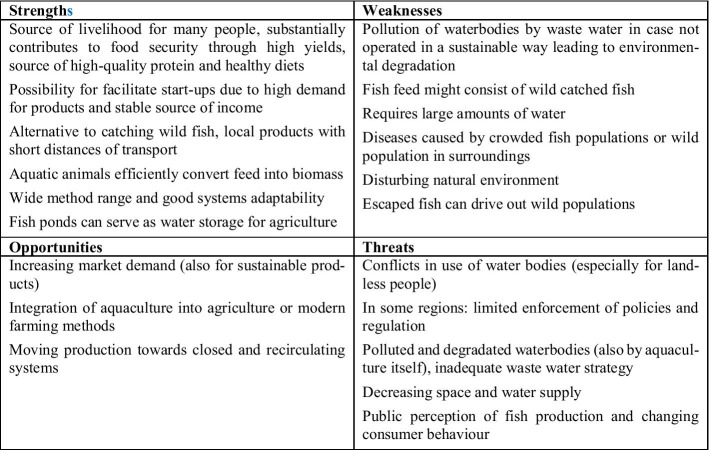
SWOT analysis of aquaculture systems

Figure 3 illustrates the results of the SWOT analysis of aquaculture systems.

**Fig. 4:**
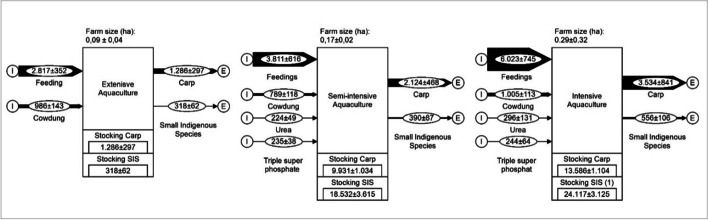
Different scales of intensification in aquaculture systems in Fulpur Upazila, Bangladesh, based on data from Mondal et al. (2012) [55]. All material flow values in kg/ha*a, all stockings in ha/a (figure: Vincent Rochell)

Figure 4 illustrates the different scales of intensification in aquaculture systems, exemplarily in Fulpur Upazila, Bangladesh based on data from Mondal et al. (2012) [55]. The figure illustrates the increased material flows of feed and additives that are needed in intensive aquaculture. While in extensive aquaculture approx. 2.8 kg/ha*a result in 1.3 kg/ha*a harvestable carp, in intensive aquaculture 6 kg/ha*a are needed to produce 3.5 kg/ha*a harvestable carp. Beside carp, small indigenous species (SIS) is harvested.

#### Integrated Plant and Animal Husbandry/Integrated Agriculture and Aquaculture

Intensive animal husbandry pollutes the environment and loses many unused nutrients through its waste such as dung, slurry, and sewage [56]. As in agroforestry, animals are used in traditional systems to recycle feed back into fertiliser in the same system (e.g. Dhesa system) [26]. Furthermore, as a result of intensive livestock farming, much of the arable land is used for growing animal feed, which is why resources are under even more pressure [57]. In Africa exist traditional agroforestry systems in which cattle graze on harvested fields and consume between 50 and 80% of the feed intake from harvest residues [46]. Sustainability and efficiency in agricultural systems can therefore be improved through the local integration of arable farming and animal husbandry [58].

Traditionally, the practice of integrated agriculture and animal husbandry exists in Asia as a rice-fish culture. Here rice cultivation and fish farming are carried out on the same area. This combination supports biodiversity, food diversity, and the use of land and water resources [26]. Thus, the systems are more resilient to climate change than monocultures. The intensification, productivity, and profitability are also strengthened in a sustainable way. The fact that phosphorus and nitrogen are more readily available in these systems also increases soil fertility and decreases the need for fertiliser [26]. In some systems, ducks are also kept in the fields to eat pests and thus improve the harvest. This results in an additional diversification of the products, and the need for fertilisers can be further reduced [26]. From an economic point of view, the integration of animals such as ducks and fish in rice fields creates improved incomes and contributes to securing food for the population [26]. According to Dalsgraad and Prein (1999) [59], an area of 800,000 hectares was cultivated with rice-fish systems in China in 1988, with 180 kg of fish being produced per hectare. In addition, the presence of fish in the fields increased rice production by at least 10%.

**Fig. 5:**
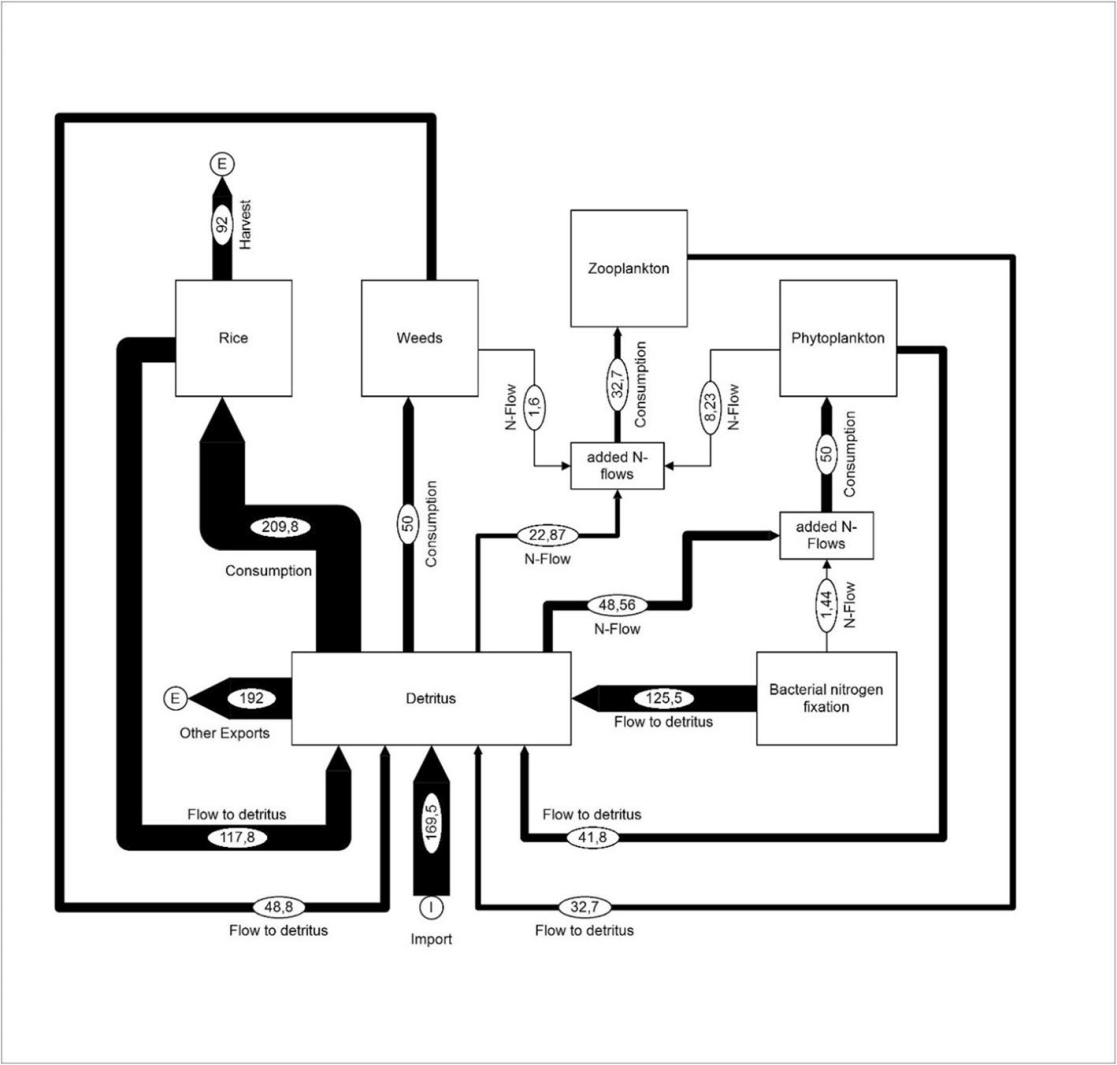
Nitrogen flow visualisation of a theoretical 1.0 ha monoculture rice farm modified according to Dalsgaard and Prein (1999) [59]. All values in kg N/ha*a (figure: Vincent Rochell)

**Fig. 6:**
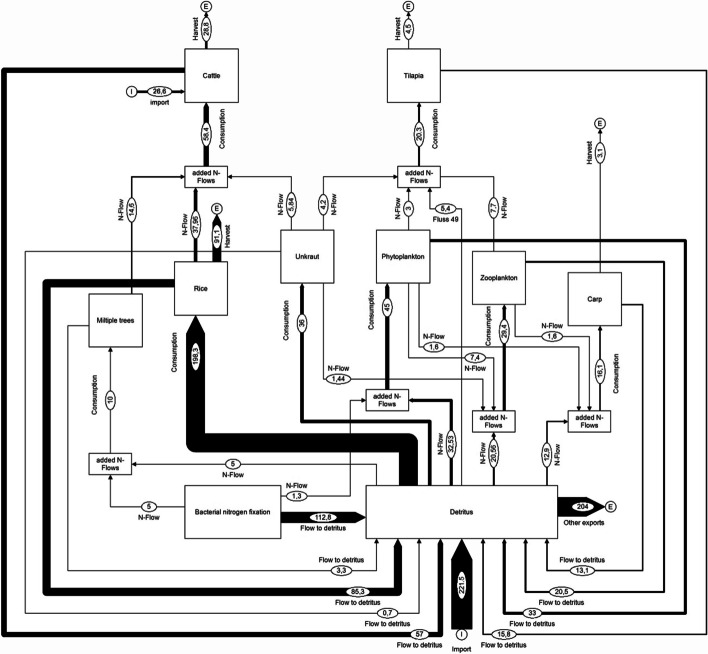
Nitrogen flow visualisation of a theoretical 1.0 ha integrated agriculture–aquaculture (IAA) farm system modified according to Dalsgaard and Prein (1999) [59]. All values in kg N/ha*a (figure: Vincent Rochell)

In order to illustrate the differences between the nutrient flows in a monoculture and in a system with integrated aquaculture and animal husbandry, this section shows material flows for nitrogen. The presentation is based on quantitative data provided by Dalsgaard and Prein (1999) [59] referring rice cultivation without fish farming and integrated rice cultivation with fish farming according to Lightfoot et al. (1993) [60]. This is a presentation of hypothetical production and consumption parameter values that relate to the individual components of the two cultivation systems and show their connections [59]. Further considered sources are Veste and Böhm (2018) [61] for tree consumption and [46] for cattle consumption. Figures 5 and 6 illustrate the structural changes in nutrient flows when a rice monoculture is converted into a diversified farm with trees and integrated aquaculture as well as cattle breeding [59]. In this model, detritus forms the basis of the soil resources [59]. It is made clear that a model with integrated fish farming leads to an increased complexity of the system. Both the number of system components and the amount of material flows have increased significantly in Fig. 6 compared to Fig. 5.

Through carp, tilapia, and cattle, a wider range of products can be ascribed to integrated fish farming and animal husbandry in a rice culture than to monoculture (Figs. 5 and 6). In addition, a significantly larger volume of nitrogen flows to the detritus can be seen. Thus, a rice cultivation system with integrated fish and animal husbandry can be assigned an increased volume of recycled nutrient flows (Fig. 6).

Another successful form of animal husbandry and aquaculture traditionally integrated into agriculture is the VAC system in Vietnam, combined horticulture with aquaculture and livestock farming. The VAC system is characterised by increased input efficiency and nutrient recycling and thus comes close to a closed system [47]. Depending on the region and its topography, farmers use an additional element that might be trees as agroforestry or rice fields. The VAC system was successfully re-introduced in Vietnam in the 1980s to cope with food scarcity.

**Fig. 7:**
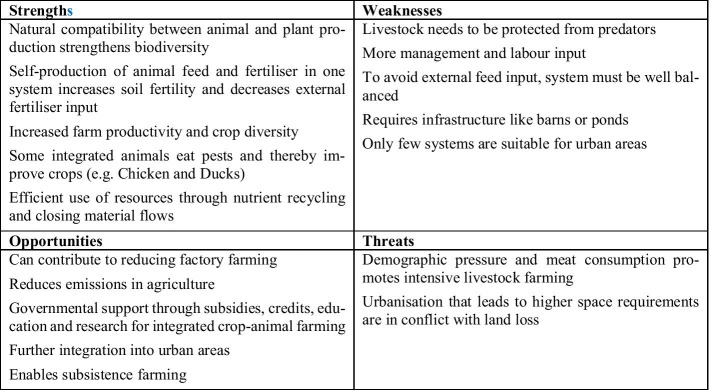
SWOT analysis of integrated plant and animal husbandry/integrated agriculture and aquaculture

Figure 7 illustrates the results of the SWOT analysis of integrated plant and animal husbandry/integrated agriculture and aquaculture.

**Fig. 8:**
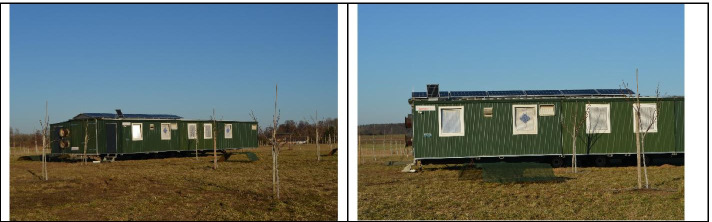
Modern integrated animal husbandry in agroforestry systems: mobile chicken farms in orchards, impressions from Germany (photo credit: Petra Schneider)

Other modern approaches combine chicken farming with the creation of orchards. The system can be considered a symbiosis between fruit and meat production that benefits from both parts: chicken ensure the loosening of and nutrition application to the soil, while orchards ensure shadow. An even further developed system is a mobile farm that takes the chickens to different places (Fig. 8).

### Modern Small-Scale Farming Systems

#### Modern Urban Farming and Urban Gardening

**Fig. 9:**
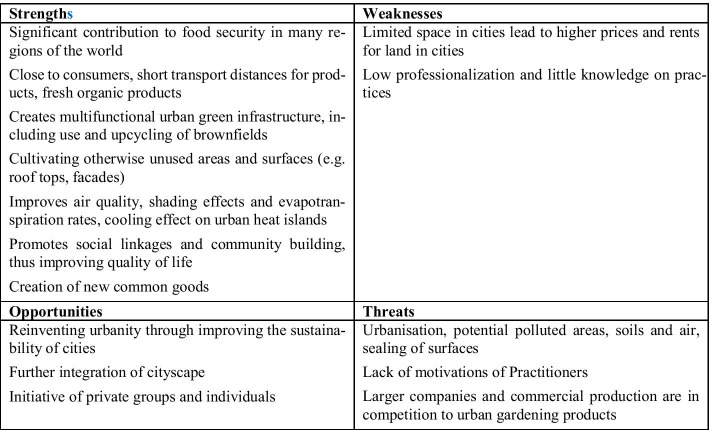
SWOT analysis of urban gardening and urban agriculture

In the global north, urban agriculture is seen as a special form of agriculture that meets the requirements of certain urban living conditions and is adapted to basic conditions in urban landscapes, such as limited but at the same time unused areas. The main difference between urban farming and urban gardening is not only the scale but also the subsidiary-oriented character of urban gardening, while urban farming is highly specialised and profit oriented. The development of new concepts and techniques is typical and can lead to new forms of food production regardless of aspects such as climatic conditions [62]. Examples of these innovative systems are hydroponics and aquaponics. Furthermore, urban agriculture can also be differentiated according to the form in which the limited available areas are used in urban environments. Furthermore, supplementary to the use of fallow land, roof areas (roof farming) can also be used for inner-city agriculture or vertical forms of cultivation (vertical agriculture) [63]. In addition to economic and ecological advantages such as short transport routes, urban agriculture and urban gardening are also said to have potential for social interactions and social change towards sustainability [61]. Especially in developing countries, where there is a high level of urbanisation, the lack of formal jobs is a motivation for urban gardening and agriculture in order to improve household incomes and to secure livelihoods. Rapid urban growth and loss of space due to development and urban land use, however, threaten urban agriculture and the resulting secure livelihoods in developing countries [33]. Figure 9 illustrates the results of the SWOT analysis of urban gardening and urban agriculture.

**Fig. 10:**
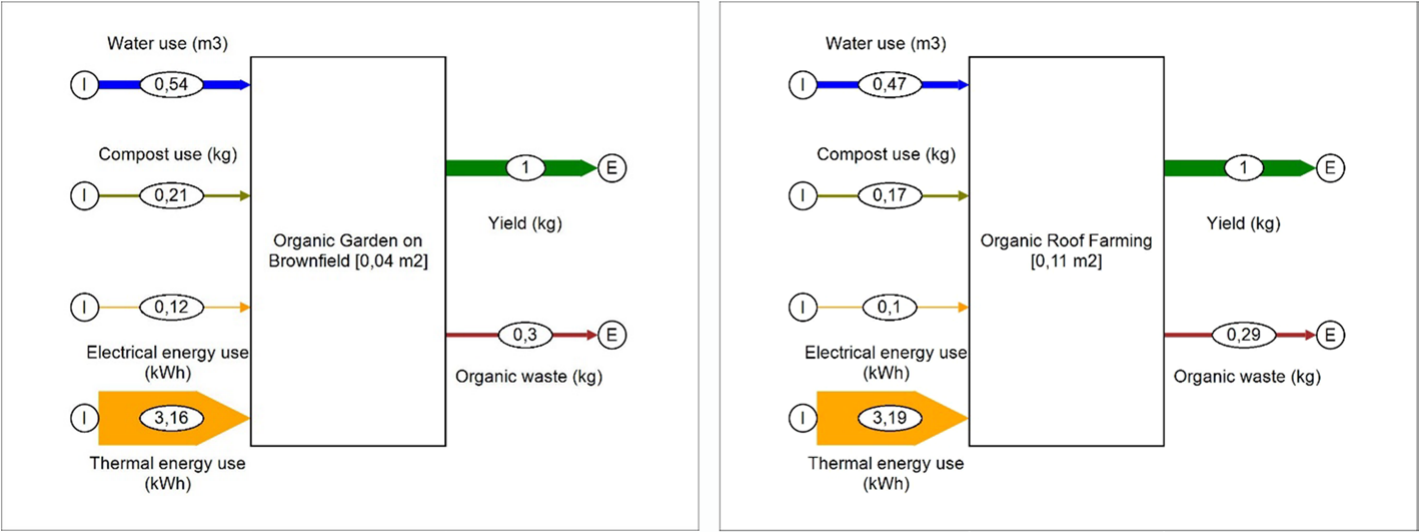
Material flows of (a) organic gardening on brownfields and (b) organic roof farming (data base: Plat, 2017) [64] (figure: Vincent Rochell)

Figure 10 illustrates the material flows of typical urban gardening systems, based on data of Plat (2017) [64], performed as (a) organic gardening on brownfields and (b) organic roof farming. The systems can be designed in a quite similar operating way, partially using greenhouses. Both systems were designed to allow year-round production with seasonal leafy greens. The assumptions are based on a location in northern Germany. This results in a partial tempering of foil greenhouses in winter.

#### Vertical Farming

**Fig. 11:**
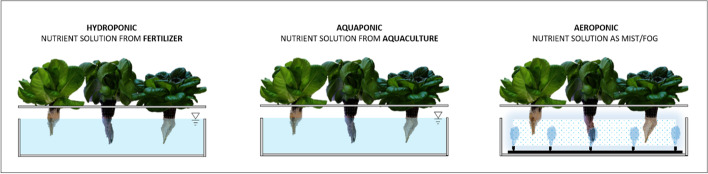
Illustration of the characteristics of hydroponic (deep water culture (DWC)), aquaponic (DWC), and aeroponic (figure source: Kay Plat)

**Fig. 12:**
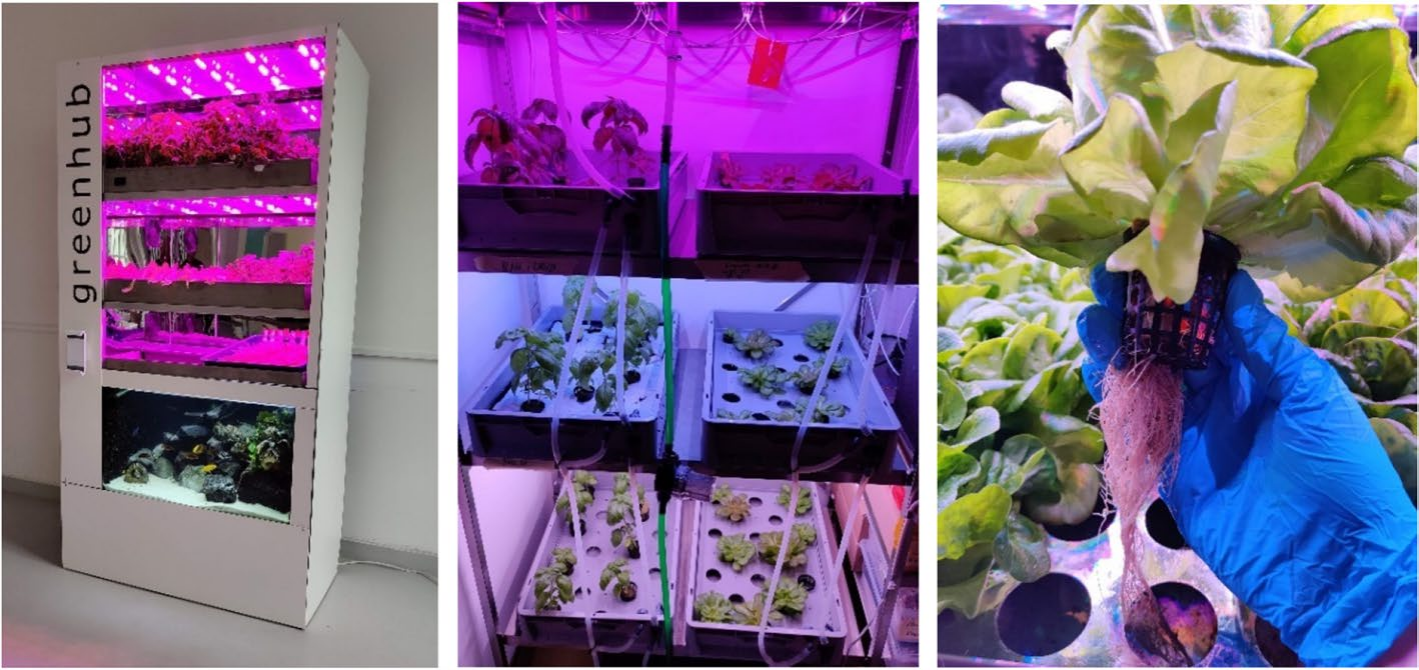
Impressions from indoor vertical farming systems at the Greenhub, Leipzig University. The left and central figures illustrate investigations with several types of lighting (photo credit: Kay Plat)

The term vertical farming covers a wide range of technologies to increase crop yields per unit of available land. This is performed by expanding crop cultivation vertically and in areas traditionally considered challenging or inaccessible for crop production [65]. Vertical farming systems come in a variety of forms, all the way from small-scale concepts to large-scale commercial approaches including simple, stand-alone two-storey or wall-mounted systems up to multi-storey high warehouses. The systems can use different soil-free cultivation methods—aquaponics, hydroponics, or aeroponics—to supply the plants with sufficient nutrients [66–68], illustrated in Fig. 11. Plants are also supplied with artificial lighting and the temperature they need to grow, as shown in Fig. 12. Vertical farming is mainly implemented as building integrated agriculture (BIA), in addition to the efficient use of space, it holds the possibility of continuous production throughout the year. Furthermore, in such environments, pests and extreme weather events do not pose a threat to the harvest and resources such as water can be more easily recycled. However, the number of cultivable plant species is reduced because slow-growing vegetables are often not profitable. Species that rely on pollination also have a disadvantage. Fast growing and fast consumable crops like leafy greens are preferred [66–68].

The significant power consumption for the operation of lighting and heating/cooling in a vertical system has an impact on economic efficiency and sustainability [66–68]. Consequently, the source of the electricity is a crucial point for the ecological footprint of the installations.

Technology-based vertical farming is still in its infancy and, according to Kozai (2018) [69], can probably double, triple, or even further increase yields in the next 10 years by realising the potential benefits, solving current problems, and taking on the challenges of the next generation of technology [69]. Nevertheless, the construction of a vertical farm in modules allows flexibility in the structure of the system and in its potential expansion [63].

In addition to indoor use in a single-purpose facility or designated areas for vertical growing systems in a multi-purpose building dedicated to growing plants year-round, vertical farming can be further categorised into edible walls, green facades, open-air, and greenhouse rooftop farming [70]. The planting of house facades and rooftops takes place in different construction methods with suitable plants. Scaffolding can be included in building planning for planting; modules in the form of vertical substrate boxes or shelving systems with a horizontal growth level can be used [67]. In most cases, this form of vertical land use is not ascribed to any agricultural use, but to an improvement in air quality, an increase in biodiversity, rainwater retention, and cooling through shading and evaporation. As Di Bonito et al. (2016) [71] reported, species as *Sedum reflexum*, *S. palmieri*, *S. acre*, *S. spurium*, *Sempervivum tectorum*, and other species are currently grown in green roofs and green facades overall in Italy. Also in Germany, sedum species is common for green roofs. However, those species are not used for food production, even green roof and facade systems would be feasible for this. The implementation of such open-air and greenhouse rooftop farms as well as green facades are competing with alternative uses, such as energy generation from solar power, which is why it is important to examine the added value and economic viability of these options [72].

However, especially the construction of shelving systems, i.e. boxes or pots on house facades, the function of garden replacement and thus the potential for vertical farming can be ascribed [67]. Recent innovations also include so-called moss walls in traffic areas to reduce air pollution.

**Fig. 13:**
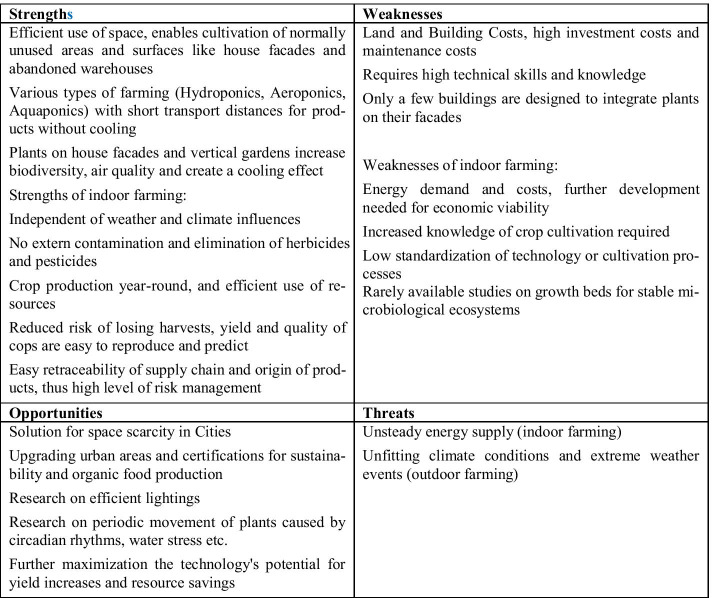
SWOT analysis of vertical farming

Figure 13 illustrates the results of the SWOT analysis of vertical farming.

**Fig. 14:**
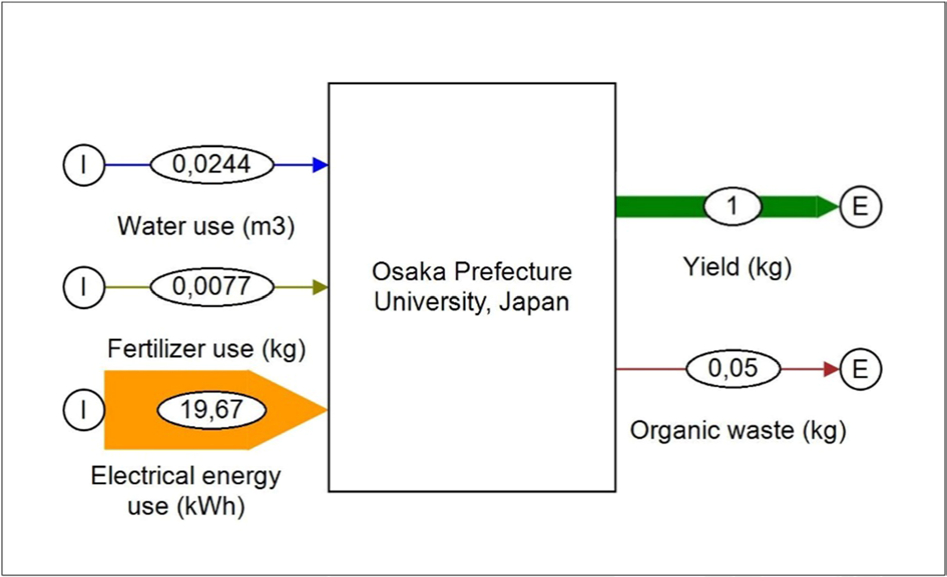
Material flow of a vertical system, based on data from a study by Ohyama et al. (2020) [73] (figure: Vincent Rochell)

Figure 14 illustrates the material flow of a vertical system based on data from a study by Ohyama et al. (2020) [73]. The values were determined for a hydroponically operated system with a floor area of 1,300 m^2^ and show the dimension of energy that is needed to operate such a system. These values have been validated in own experiments with a comparable model setup in cabinet format at the University of Leipzig (Greenhub), performed by the authors.

#### Hydroponics-Aquaponics-Aeroponics

The cultivation of plants without soil is called hydroponics. The plants are only supplied with water and nutrient solutions to the roots. Depending on the system, growing media such as rock wool, peat, pumice, or coke fibre can also be used [74]. Hydroponics also has great potential for automation, as parameters such as temperature, nutrient supply, and pH values can be determined and automatically controlled in real time using comprehensive measurement and control techniques [74, 75]. Hydroponics is divided into open and closed systems. In open systems, the nutrient solution is not reused. Instead, it is discharged into soil or surface waters or used in open field cultivation. In closed systems, the excess nutrient stream is recycled by collecting it after its first use and returning it to the system [74, 75]. Soil free systems such as hydroponics offer gardeners with limited space available for conventional horticulture the opportunity to use other spaces in addition [76]. The most important approaches for growing with hydroponics are the Deep-Flow-Technique (cultivation of plants on floating or hanging supports) and Nutrient-Film-Technique. In recent years, aeroponic systems have also been used that spray the nutrient solution onto the roots of the plant [77]. Aeroponics is mainly aimed at smaller horticulture. Plants are carried by plastic plates or styrofoam, which rest on growing boxes. The boxes are thus closed, and pipes with nutrient solution run through them. Inside the box module, a hanging root system develops that is sprayed with nutrient solution from the pipes by sprinklers. Some systems use vibrating plates. This creates micro-water droplets that form a vapour that condenses on the roots. Leachate can be collected at the bottom of the module and reused. Due to the high investment and management costs, this system is not yet widely used [74].

**Fig. 15:**
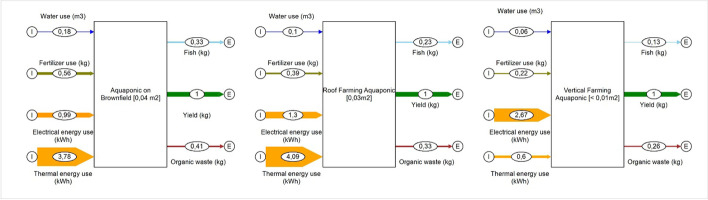
Material flows of potential aquaponic system applications, on brownfields, roof farming, and vertical farming (data source [64]) (figure: Vincent Rochell)

Aquaponics is a process that combines the techniques of rearing fish in aquaculture and the cultivation of useful plants using hydroponics. An aquaponics system is a combination of a circuit system for fish production and a hydroponic system for growing plants, for example for vegetables and herbs. The system works by using the excrement from fish farming as nutrients for plants. This is usually done automatically using pump systems. The nutrient input required for plant rearing is thus carried out through the fish feed. Figure 15 illustrates the material flows of one loop potential aquaponic system applications, on brownfields, roof farming, and vertical farming, based on data of Plat (2017) [64].

**Fig. 16:**
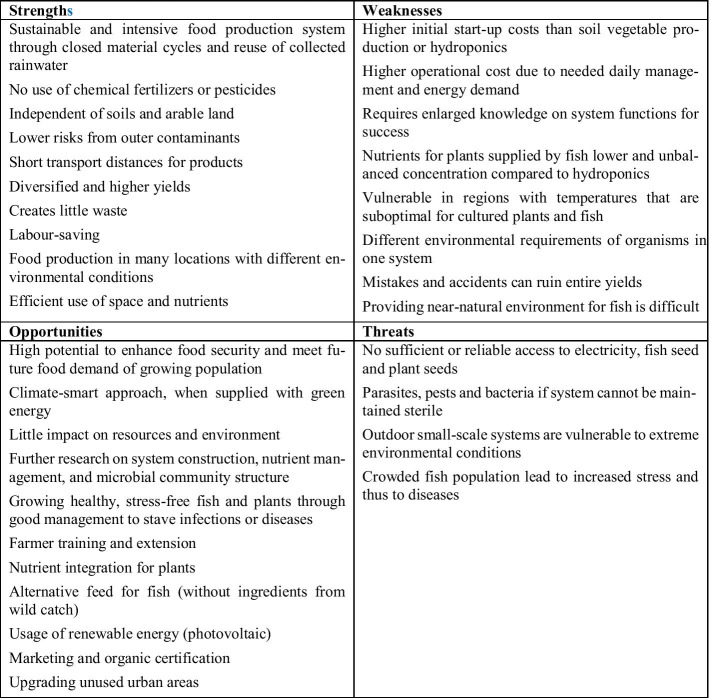
SWOT analysis of aquaponics

The calculations were made with the assumption of climatic conditions from the north of Germany. It becomes obvious that the by far highest input is needed from energy, which forms a challenge for an economically feasible operation on the long term. Figure 16 illustrates the results of the SWOT analysis of aquaponic.

#### Permaculture

The term permaculture originates from the literature on permanent agriculture. According to Ferguson and Lovell (2014) [78], it can be assumed that at the beginning of the twentieth century, the word “permanent” embodied a meaning comparable to that of the term sustainability in relation to agriculture [78–80]. Therefore, permaculture is a sustainable agricultural method that avoids the use of fossil fuels, artificial fertilisers, and pesticides [81]. For this purpose, patterns and elements of the natural ecosystem are imitated and optimised [82].

**Fig. 17:**
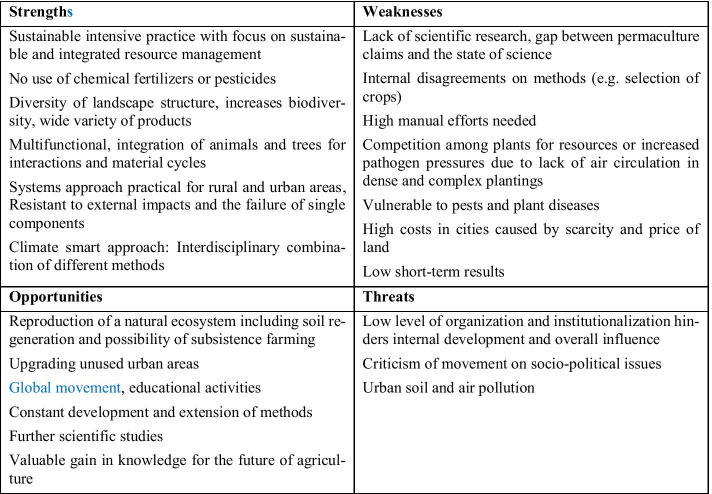
SWOT analysis of permaculture

In addition to the basic practice of observing and imitating the natural compositions and processes of an ecosystem or optimising areas that are beneficial for humans, multifunctionality is also always sought. For example, hedges can fulfil a protective function and at the same time provide habitats for animals. Permaculture should also be more resilient (if, for example, a component fails) and, if possible, have closed material cycles. Composting is implemented, and animal manure and organic fertilisers are used. Physical barriers are created for pest control, and species-rich ecosystems are sought in order to use natural enemies of pests for plant protection [79–84]. Overall, permaculture has a lot in common with agroecology, agro-forestry, and traditional and indigenous land uses [82]. It often bundles techniques and practices from these areas and gives recommendations and evaluations for successful application. Here, multi-year polycultures as well as annual vegetables play an important role [78–84]. Elements used from forestry are, for example, alley cropping and the use of trees as wind protection. The idea of the forest garden is also important, as it is said to have the potential to replicate a natural ecosystem. Animal husbandry can also be integrated and used multifunctionally. Various poultry provide food, contribute to pest control, and produce natural manure. Larger farm animals can also be kept out of pastures [81]. Using various techniques such as cover cropping and the reuse of grey water, water consumption should be kept as low as possible. Figure 17 illustrates the permaculture SWOT analysis results.

Well-known examples of the use of permaculture are still rare. Nevertheless, further research could be very valuable for the future of agriculture [83]. The Bec Hellouin Farm Model in France is a permaculture-inspired project that operates without mechanical help, pesticides, or mineral fertilisers. The farm has a total size of approximately 1 ha of which 0.1 ha are cultivated [85]. This area is cultivated by a total of 7 people. In cooperation with various scientific institutions, it was possible to confirm high productivity in a small area. In 2015, products with a market value of € 55,000 were sold on an area of 1000 m^2^. Organic vegetable cultivation in France otherwise generates an average yield of around € 30,000 per 10,000 m^2^ [86]. In addition, improved soil quality can be assumed [85].

Even the permaculture approach is still in scientific discussion, it can be considered an ecological engineering methodology in the field of food production and human settling in a whole that uses several of the discussed elements like agroforestry to create resilient, circular, and climate smart cultivation approaches.

## Discussion and Conclusions

Circular approaches for water and nutrient management have a long history in traditional food production methodologies even the efficiency compared to industrial agriculture is much lower. However, there lies a substantial potential of circular flux management in small-scale food production that could be transposed to a larger scale also, particularly in terms of agroforestry and integrated plant and animal husbandry or integrated agriculture and aquaculture. These methodologies are based on a systems approach that copies and adopts natural cycles and in way represent applied ecological engineering. As consumers started to change their nutritional behaviour during the COVID-19 pandemic, integrated agroecology might provide prospects in a Post-Covid Era.

Having in view the huge environmental impacts of industrial agriculture, there is a need to transform such systems into a more ecological form. In the small-scale form, we might call this circular gardening, on the larger scale agroecology. Future efforts must be put into the permaculture subject to foster the integration of the wide range of circular and ecological food production approaches to develop an integrated agroecology. Agroforestry elements should be a substantial part of each integrated agroecological system to ensure sustainability in an agro-based bioeconomy. In this way, small-scale food production will be part of a bioeconomy for sustainable development [87] and holds a large potential for food security and the future implementation of the water-energy-food security nexus [88]. In practice, the water-energy-food security nexus can be only implemented through an integrated management of material fluxes and of resources, particularly a circular flux management, and the material flow analysis is the basic tool to identify potential gaps in the material flows. Having identified the gaps, mitigation strategies shall be developed, as can be currently observed in the renaissance of traditional food generation approaches in the Global North, like, for instance, agroforestry.

**Fig. 18:**
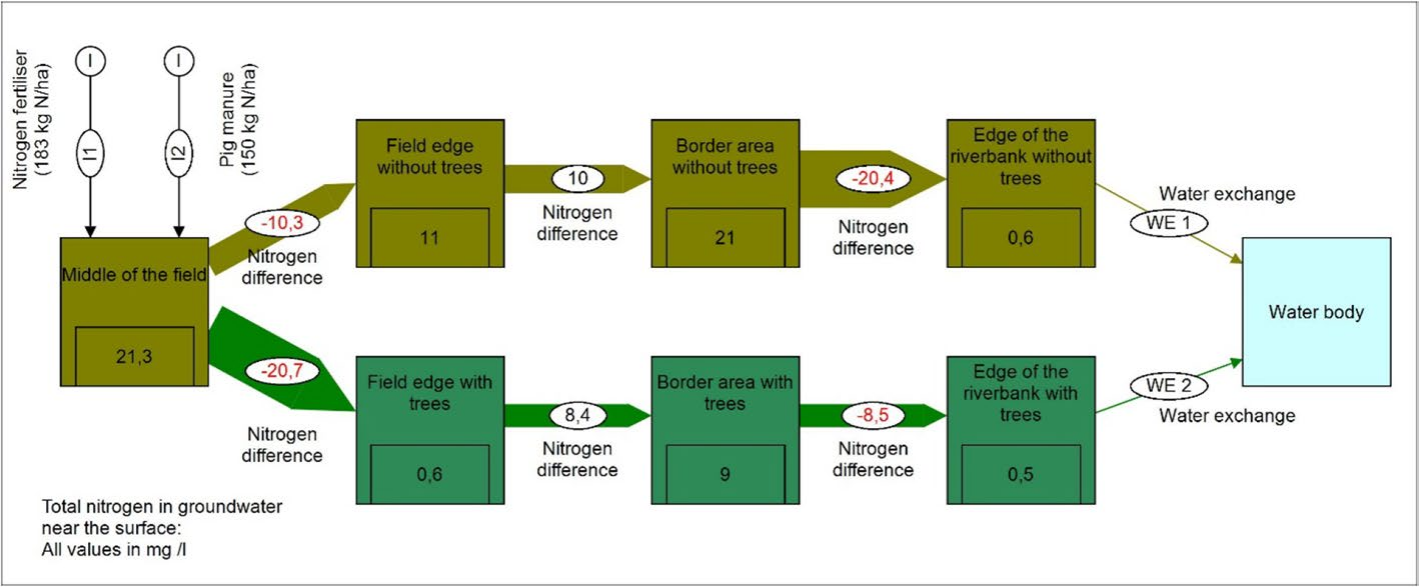
Material flows of total nitrate in groundwater near the surface in 2017: red flows without agroforestry use, green flows with agroforestry use, based on data of Tsonkova and Böhm (2020) [44] and Veste and Böhm (2018) (figure: Vincent Rochell)

Beside the ongoing traditional agroforestry use in the arid and semi-arid regions like Africa, Spain, and Greece [89], there are initiatives for a renaissance of agroforestry in the temperate regions of the Global North, like in Germany (for instance [41, 43, 44, 61, 89]. The results show supplementary benefits to food production. Grünewald et al. 2007 [41] proofed that agroforestry systems might be applied for the production of woody biomass for energy transformation purposes, e.g. to replace fossil fuels with renewable energy sources. Furthermore, there was observed a substantial benefit for improvement of the groundwater quality in terms of existing high nitrogen concentrations, as reported by Tsonkova and Böhm (2020) [44] and Veste and Böhm (2018) [61]. Figure 17 illustrates exemplarily the material flows of total nitrogen in groundwater near the surface from an intensively cultivated field towards a bypassing watercourse (red flows without agroforestry use, green flows with agroforestry use as riparian buffer strips). The figure underlines that agroforestry can support to mitigate high nutrient concentrations. Furthermore, those systems support substantially the water balance and in this way climate change adaptation [82, 86]. In this way, agroecological systems are multifunctional systems (Fig. 18).

Technical forms of cultivation (aquaponics systems, vertical farming, etc.) are usually associated with high investment costs and require a lot of effort in management. In these cases, small scale might be considered very small (container size) or aquaponics systems with IBC tanks in the garden. The approach to combine traditional farming methods with modern ones will play an important role in the future to get advantages from both approaches. It can be seen that depending on climatic conditions, land availability, investment, and labours, the used forms of cultivation are different. For planned projects, site-specific conditions must be verified in advance, and a suitable method must then be selected. Furthermore, the elaboration of a life cycle assessment is indicated to assess all types of potential resource flows, consumptions, and impacts for the whole system including the externalised effects. The source of the energy supply plays a substantial role in the long-term sustainability and LCA assessment of each system.

## Data Availability

The used data are available from the authors upon request.

## Code Availability

The used STAN software is freeware, available under https://www.stan2web.net/.
